# Survival, recurrence and toxicity of HNSCC in comparison of a radiotherapy combination with cisplatin versus cetuximab: a meta-analysis

**DOI:** 10.1186/s12885-016-2706-2

**Published:** 2016-08-26

**Authors:** Jingwen Huang, Jing Zhang, Changle Shi, Lei Liu, Yuquan Wei

**Affiliations:** 1 Department of Medical Oncology, Cancer Center, West China Hospital, Sichuan University, Chengdu, China; 2 State Key Laboratory of Biotherapy and Cancer Center, West China Hospital, West China Medical School Sichuan University, Chengdu, China; 3 West China Medical School, West China Hospital, Sichuan University, Chengdu, China

**Keywords:** HNSCC, Oropharynx, HPV, Cisplatin, Cetuximab, Radiotherapy, Prognosis, Recurrence, Adverse event

## Abstract

**Background:**

Cisplatin-based treatment has been considered the standard treatment regimen of HNSCC. Cetuximab is an emerging target therapy that has potential therapeutic benefits over cisplatin. Nevertheless, curative effects of cisplatin-based chemoradiotherapy (CRT) versus cetuximab-based bioradiotherapy (BRT) are still controversial.

**Methods:**

Potentially eligible studies were retrieved using PubMed, Embase and Medline. Basic characteristics of patients and statistical data were collected. A meta-analysis model was established to compare CRT and BRT.

**Results:**

Thirty-one eligible studies and 4212 patients were found. The pooled HRs with 95 % confidence intervals (CIs) for OS and PFS were 0.32 [0.09, 0.55] and 0.51 [0.22, 0.80], respectively, and both were in favor of cisplatin. However, 3-year survival and recurrence analysis of the subgroups showed no differences between the two groups (*p* > 0.05). In subgroup analysis, oropharyngeal primary tumors exhibited improved results by cetuximab with a pooled HR of 1.56 [1.14, 2.13] for PFS. Additionally, the HPV+ status was a significant factor in positive outcomes with cetuximab with a pooled HR of 1.12 [0.46, 2.17] for OS.

**Conclusion:**

Long-term use of BRT showed no significant difference compared with CRT, and both arms showed different aspects of toxicity. In subgroup analysis, taking the effects of treatment and adverse events into consideration, cetuximab plus radiation may show superior responses regarding OS and PFS in patients who have HPV+ or primary oropharyngeal HNSCC, respectively, but physicians should administer them with caution.

## Background

Squamous cell carcinoma of the head and neck (HNSCC) consists of cancers arising from the oral cavity, pharynx and larynx and comprises approximately 5 % of all cancers worldwide. The global incidence is increasing by half a million and causing more than 350,000 deaths every year [1, 2]. A limited number of patients with locally advanced disease are suitable for potentially curative surgery or definitive radiotherapy. Patients who are not candidates for surgery or definitive radiotherapy may receive chemotherapy plus radiation or systemic chemotherapy alone [3].

Cisplatin-based chemoradiotherapy is now considered to be the established standard, first-line chemotherapy to treat patients with locally advanced HNSCC [69]. Many large randomized studies and meta-analyses have demonstrated that cisplatin-based concurrent chemoradiotherapy regimens provide significantly higher response rates than radiotherapy alone [4, 5].

Epidermal growth factor receptor (EGFR) seems to be critical to cancer cell growth and proliferation, and the function of EGFR in these two settings appears to be different [6, 7]. Head and neck cancer cells exhibit this difference compared to normal cells without exception [8]. In addition, EGFR expression was markedly increased or over-expressed in HNSCC compared to normal tissue, which has been shown to be an independent prognostic factor for poor survival [9]. Thus, EGFR inhibitors have become a burgeoning strategy in anti-tumor treatment. To date, several monoclonal antibodies targeting EGFR have been successfully used in clinical practice with significant effects. Improved loco-regional control and prolonged survival time have already been achieved in lung and gastro-intestinal cancers [10–12].

Cetuximab, an EGFR-targeting monoclonal antibody, is the first targeted therapy to show therapeutic benefit in head and neck cancer [13] and received FDA approval for use in treating HNSCC in 2006 [14, 15]. The Bonner trial showed impressively increased survival outcomes and loco-regional control rates when comparing cetuximab plus radiation versus radiation alone [16]. The Merlano trial exhibited a promising treatment response from adding cetuximab to standard chemotherapy, with limited toxicity [17]. Clinical trials have shown that the addition of cetuximab to traditional treatment regimens (e.g., cisplatin plus radiation) could improve survival outcomes [18, 19]. However, this combination may lead to increased treatment-related toxicity and increased cost, and the administration of multiple drugs may worsen quality of life.

Hence, we conducted a meta-analysis with the aims of gathering outcomes from clinical trials and obtaining a larger sample size to compare the curative effects between the administration of cisplatin-based chemoradiotherapy (CRT) or cetuximab-based bioradiotherapy (BRT) with regards to survival results, loco-regional control or distant metastasis (failure), and treatment-related adverse effects in patients with HNSCC.

## Methods

### Search strategy

PubMed, Embase and Medline were searched on Mar 13, 2016. The following keywords were used to retrieve articles and abstracts: head and neck squamous cell carcinoma (HNSCC), cancers of larynx, cancers of oral tongue, cancers of oropharynx, cancers of laryngopharynx, cetuximab, cisplatin and radiotherapy.

### Study selection and inclusion/exclusion criteria

Titles and abstracts were reviewed in all of the searched studies, and full texts were reviewed in potentially eligible studies according to our inclusion criteria. To avoid duplicated data, when more than one trial was completed with crossed data in a single center, only the largest most updated trials were included.

In our meta-analysis, we used the following inclusion criteria: (1) studies containing patients with locally advanced HNSCC, including the following: cancers of the larynx, cancers of the oral tongue, cancers of the oropharynx, or cancers of the laryngopharynx; (2) studies comparing the administration of cisplatin-based chemotherapy versus cetuximab-based biotherapy; and (3) studies with available data regarding survival outcomes of patients included in the clinical trials. On the other hand, studies were excluded based on the following criteria: (1) articles that consisted of in vitro studies or were review articles; (2) studies with duplicated data, meaning that one analysis that had several articles reporting updated outcomes; and (3) studies containing metastatic and/or recurrent disease.

### Data extraction

The following two investigators reviewed all of the articles independently: Huang JW and Shi CL. Any discrepancy was discussed until reaching a consensus. The data were independently extracted from eligible studies by two investigators (Huang JW and Shi CL), and then, the obtained data were integrated. The primary data consisted of HRs with a 95 % confidence interval (CI) or event/total patient numbers regarding survival outcomes, including OS and/or PFS and the recurrence rates, such as loco-regional and/or distant recurrence of disease in patients from cetuximab cohorts and cisplatin cohorts.

The additional data obtained from the studies included the first author, publication year, patient source (region), median age, percentage of each sex, TNM stage at diagnosis, treatment regimens, tumor site (%), survival outcomes, recurrence rates, type of study, toxicity N (G3 ~ 4) in CRT vs. BRT groups, and attitude of the original studies. The statistical data for acquiring logHR and SE were also obtained, including HR with a 95 % CI, Kaplan–Meier survival curves with p values, and response rates of the over-expression cohort compared to the normal/lower expression cohort [20].

### Statistical methods

logHR and SE were required in our analysis. Some of the original papers provided logHR and SE directly, whereas other studies did not. As mentioned above, we utilized other data to calculate these values using methods developed by Parmar et al. (1998) [21], Williamson et al. (2002) [22], and Tierney et al. (2007) [23]. The logHRs and SEs were calculated with the methods described earlier when 1) there was a HR with 95 % CI or 2) there was a p value for the log-rank test with the Kaplan–Meier survival curve.

Hazard ratio (HR) was used as the measure index to describe the survival outcomes and disease control rates between the BRT arm and CRT arm (we considered the cisplatin regimen as the standard regimen). As a result of the analysis of survival in patients, a significant outcome was defined by a *p* value < 0.05, while a *p* value > 0.05 indicated no significant difference between the two comparison arms. Pooled HRs > 1 combined with *p* < 0.05 indicate a narrow difference between the two groups, and the cetuximab arm showed higher event incidences. In contrast, pooled HRs < 1 indicated a lower incidence of events in the cetuximab cohort. Furthermore, pooled HRs > 2 or <0.5 denote a significant result. We use the term “positive” to indicate a better outcome related to cetuximab treatment and “negative” to indicate an absence of correlation between the two comparison arms or better outcomes in the cisplatin arm.

In terms of heterogeneity, values of *p* < 0.10 or I^2^ > 50 % represent heterogeneity existing in the pooled HRs (Higgins et al., 2003) [24]. When homogeneity was minimal (*p* ≥ 0.10, I^2^ ≤ 50 %), a fixed-effects model was applied for secondary analysis; otherwise, a random-effects model was used. All of the earlier calculations and publication bias were measured using the Begg’s funnel plot, which was performed by STATA 11.0 (STATA Corporation, College Station, TX). This calculation for the current meta-analysis was performed using REVIEW MANAGER (version 5.0 for Windows; the Cochrane collaboration, Oxford, UK).

The sensitive analysis, which aims to test for the heterogeneity of all of the included studies and to determine if heterogeneity arose from any single study, was performed by STATA 11.0 (STATA Corporation, College Station, TX). In the analytic figure, an absence of heterogeneity is indicated by the containment of the studies within the constricted interval (defined between lower CI limit and Upper CI limit), while the existence of a single study far outside the confidence interval indicates that the heterogeneity is due to that individual study.

## Results

### Eligible studies

**Fig. 1:**
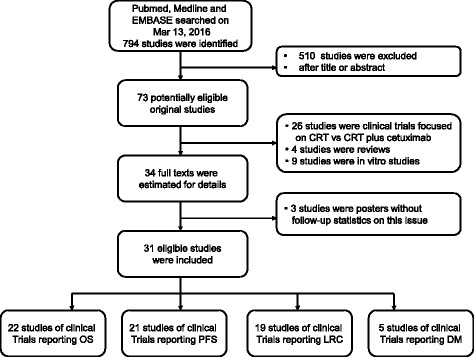
Selection of Studies

We initially obtained 794 studies from PubMed, Embase and Medline. After reviewing these abstracts, 73 potentially relevant studies were identified as candidates for a full-text review. We excluded 42 studies for the following reasons: twenty-one were clinical trials focused on CRT vs. CRT plus cetuximab, four were reviews, three were posters without follow-up statistics on the studies, and seven were in vitro studies (Fig. 1).

Finally, we enrolled 31 eligible articles containing survival outcomes [25–50]. These eligible studies were published from 2008 to 2016 and included a total of 4212 patients, ranging from 24 to 421 patients per study (median, 126). The basic clinical characteristics of patients and other useful information are shown in Table 1.

**Table 1:** Basic characteristics of included studies

Author	Type of study	Region	Age		Stage	Female (%)		Therapy regimens	HPV statue (+)		Survival
			Cisplatin	Cetuximab		Cisplatin	Cetuximab		Cisplatin	Cetuximab	
Vermorken JB	RS	America	CRT: 57.8	BRT: 57	Stage III/IV	NR		RT-CIS VS. RT-CET	NR		2-yrOS 1-yr PFS
Caudell JJ	RS	America	CRT: 55	BRT: 54	Stage III/V	BRT: 20.7 %	CRT: 22.3 %	CCRT VS. Concurrent RT+CET	NR		1-yr OS 2-yr OS
L.D. Koutcher	RS	America				NR		RT-CIS VS. RT-CET	16 (42 %)	8 (35 %)	30-mon PFS 30-mon OS
Jensen AD	RS	Germany	CRT: 38	BRT: 38	Stage III/IV	NR		RT-CIS VS. RT-CET	NR		2-yr OS 2-yr PFS 2-yr L-PFS 2-yr D-PFS
Koutcher L	RS	America	CRT: 56	BRT: 66	Stage III/IV	CRT:17 (13.6 %)	BRT:11 (22.5 %)	RT-CIS VS. RT-CET	NR		2-yr FFS 2-yr OS 2-yr LRC
Beijer, Y.J.	RS	Netherland	Primary: 56	Primary: 64	Stage II-IV	CRT Primary: 37	CET Primary: 43	RT-CIS VS. RT-CET	NR		1-yr OS 1-yr DFS 2-year OS 2-yr DFS LRR
			Adjuvant: 59	Adjuvant: 56		CRT Adjuvant: 36	CET Adjuvant: 36				
Ley J	RS	America	CRT: 55	BRT: 62	Stage III/IV	CRT: 16.7	BRT: 34.5	RT-CIS VS. RT-CET	NR		3-yr DSS 3-yr LRR
Ye AY	RS	Canada	CRT: 57	BRT: 62	Stage III/IV	CRT: 17	BRT: 14	RT-CIS VS. RT-CET	NR		3-yr OS 3-yr DFS 3-yr LRC
Pajares B	RS	Spain	p16 Negative: 59	p16 positive: 57	Stage III/IV	p16 Negative:7	p16 Positive: 6	RT-CIS VS. RT-CET	10 (18 %)	8 (15 %)	2-yr OS 2-yr DFS 2-yr LRR
Lefebvre JL	Phase II RCT	France	CRT: 57.5	BRT: 57.8	Stage II-IV	CRT: 13.3	BRT: 1.7	RT-CIS VS. RT-CET	NR		18-mon OS 18-mon LRR 36-mon OS
M. Ghi	RCT	Italy	60		Stage III/IV	80.5		CCRT VS. Cet+RT	NR		3-yr OS 3-yr PFS
N. Riaz	RCT	America	NR		NR	NR		CCRT VS. Cet+RT	24 (56%)	11 (75%)	NR
Hu MH	RCT	Taiwan	CRT: 55	BRT: 78	Stage III/IV	CRT: 3.4	BRT: 3.7	CCRT VS. Cet+RT			LRR 3-yr RFS 3-yr OS DM
Levy A	RCT	Germany	CRT: 58	BRT: 60	Stage III/IV	CRT: 20	BRT: 23	CCRT VS. BRT	NR		2-yr OS 2-yr LRC 2-yr DM
Tang C	RCT	America	CRT: 58	BRT: 73	Stage I-IV	CRT: 10	BRT: 1	CCRT VS. Concurrent Cet+RT	NR		2-yr LRC 2-yr EFS 2-yr OS
Fayette J	RS	France	56		Stage III/IV	10		CCRT VS. Concurrent Cet+RT	NR		5-yr OS 5-yr DFS
Huang J	RS	Japan	CRT: 55	BRT: 77	Stage III/IV	IMRT/ cisplatin: 13	IMRT/ cetuxima:19	IMRT/CIS VS. IMRT/CET	NR		LRC DM OS CSS
Shapiro LQ	RS	America	NR		stage II-IV	CRT:13.1	BRT: 22.4	IMRT/CIS VS. IMRT/CET	NR		4-yr OS 4-yr LRF
M.R. Kanakamedala	RS	America	53		NR	NR		RT-CIS VS. RT-CET	NR		LRC 3-yr OS 2-yr PFS
Riaz N	RS	America	NR		NR	NR		RT-CIS VS. RT-CET	NR		NR
Riaz N	RS	America	NR		Stage III/IV	CRT: 21	BRT: 22	RT-CIS VS. RT-CET	31 (86 %)	17 (74 %)	NR
Peddi P	RS	America	CRT: 55	BRT: 61	Stage III/IV	CRT: 26.7	BRT: 29.7	CCRT VS. Concurrent RT-CET	NR		2-yr OS 2-yr PFS
S.L.Galper	RS	America	CRT: 58	BRT: 71	NR	NR		RT-CIS VS. RT-CET	NR		NR
D.Borchiellini	RS	France	CRT: 56	BRT: 57	NR	CRT 16 %	BRT 8 %	RT-CIS VS. RT-CET	NR		NR
Lorraine Walsh	RS	Ireland	CRT: 57.5	BRT: 63	Stage III/IV	CRT : 9 %	BRT : 11.8 %	RT-CIS VS. RT-CET	NR		NR
Stefano Maria Magrini	RCT	America	CRT: 67.5	BRT: 61	Stage III/IV	CRT: 31 %	BRT:26 %	RT-CIS VS. RT-CET	NR		2-yr OS
Tobin J. Strom	RS	America	CRT: 58	BRT: 62	Stage III/IV	CRT: 16.2	BRT: 5.3	RT-CIS VS. RT-CET	43.4 %	41.2 %	2-yrOS
Nadeem Riaz	NR	America	CRT: 118 < 71 7 >71	BRT: 38 < 71 11>71	NR	CRT : 21 %	BRT: 22 %	RT-CIS VS. RT-CET	86	74	3-yr LRC 3-yr OS 3-yr PFS

*CRT* cisplatin-based chemoradiotherapy, *BRT* cetuximab-based bioradiotherapy, *RT-CIS* radiation plus cisplatin, *RT-CET* radiation plus cetuximab, *CCRT* concurrent chemoradiotherapy, *yr* year, *mon* month, *HPV* Human papillomavirus, *RS* retrospective study, *RCT* randomized controlled study, *OS* overall survival, *PFS* progression free survival, *L-PFS* local progression free survival, *D-PFS* distant progression free survival, *FFS* failure-free survival, *LRR* locoregional recurrence, *DFS* disease free survival, *DSS* disease specific survival, *LRC* locoregional control, *RFS* relapse-free survival, *DM* distant metastasis, *EFS* event-free survival, *CCS* cause-specific survival, *CAD/CVD* coronary artery disease/cardiovascular disease, *COPD* chronic obstructive pulmonary disease, *PNS* peripheral nervous system, *NR* not reference

### Comparison between cisplatin-based and cetuximab regarding overall survival

**Fig. 2:**
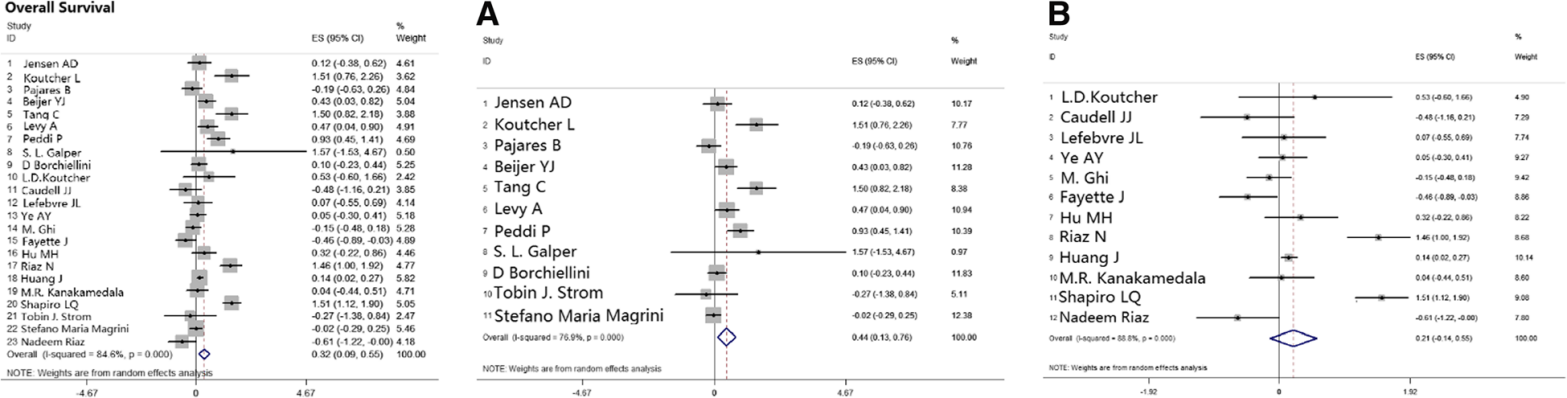
Meta-analysis estimated OS comparing cisplatin-based chemoradiotherapy versus cetuximab-based bioradiotherapy. (**a**) subgroup of estimation of 2-yr OS; (**b**) subgroup of estimation of 3-yr OS. OS, overall survival

Twenty-three settings of accommodated data showed patients’ overall survival (OS). In these trials, patients were scheduled to receive cisplatin-based chemotherapy plus radiation or cetuximab single agent plus radiation. The pooled HRs to compare OS between the two groups showed better outcomes with cisplatin-based therapy and the mathematic value is 0.32 [0.09, 0.55], *p* = 0.006 (Table 2; Fig. 2).

**Table 2:** Pooled HRs (95 % Cl) comparing survival outcomes and recurrence between BRT & CRT

Comparison	Survival outcome	Study N.	Model	HR (95 % Cl)	*P* value	Heterogeneity (p ,I^2^)	Conclusion
BRT vs. CRT	OS	23	Random	0.32 [0.09, 0.55]	0.006	*P* < 0.00001; I² = 84.6 %	Positive
BRT vs. CRT	OS for 2-yr	11	Random	0.44 [0.13, 0.76]	0.006	*P* < 0.0001; I² = 76.9 %	Positive
BRT vs. CRT	OS for 3-yr	12	Random	0.21 [-0.14, 0.55]	0.241	*P* < 0.00001; I² = 88.8 %	Negative
BRT vs. CRT	PFS	21	Random	0.51 [0.22, 0.80]	0.001	*P* < 0.00001; I² = 90.1 %	Positive
BRT vs. CRT	PFS for 2-yr	10	Random	0.56 [0.20, 0.92]	0.002	*P* < 0.00001; I² = 88.2 %	Positive
BRT vs. CRT	PFS for 3-yr	11	Random	0.45 [-0.05, 0.95]	0.076	*P* < 0.00001; I² = 91.8 %	Negative
BRT vs. CRT	Locoregional control	19	Random	0.49 [0.14, 0.85]	0.007	*P* < 0.00001; I² = 91 %	Positive
BRT vs. CRT	Locoregional control for 2-yr	9	Random	0.63 [0.09, 1.17]	0.023	*P* < 0.00001; I² = 83 %	Positive
BRT vs. CRT	Locoregional control for 3-yr	10	Random	0.06 [-0.40, 0.52]	0.808	*P* < 0.00001; I² = 93.3 %	Negative
BRT vs. CRT	Distant control	5	Random	0.25 [0-0.06, 0.56]	0.118	*P* < 0.00001; I² = 88.3 %	Negative
BRT vs. CRT	OS for oropharynx	7	Random	0.13 [-0.03, 0.89]	0.743	*P* < 0.00001; I² = 84.8 %	Negative
BRT vs. CRT	PFS for oropharynx	3	Random	1.56 [1.14, 2.13]	0.006	*P* < 0.00001; I² = 96 %	Positive
BRT vs. CRT	Locoregional control for oropharynx	6	Random	1.75 [0.6, 5.26]	0.31	*P* < 0.00001; I² = 89.1 %	Negative
BRT vs. CRT	OS for HPV+	5	Fixed	1.12 [0.46, 2.17]	0.015	*P* = 0.22; I² = 38 %	Positive
BRT vs. CRT	PFS for HPV+	5	Random	0.80 [0.38, 1.67]	0.55	*P* < 0.00001; I² = 92 %	Negative
BRT vs. CRT	Locoregional control for HPV+	5	Random	1.17 [0.69, 2.00]	0.56	*P* = 0.01; I² = 71.1 %	Negative

*CRT* cisplatin-based chemoradiotherapy, *BRT* cetuximab-based bioradiotherapy, *N* number, *OS* overall survival, *PFS* progression-free survival, *CI* confidence interval, *HR* hazard ratio, *yr* year

#### Subgroup analysis

As survival outcomes were largely influenced by time of observation, we categorized OS outcomes by year of estimation: 2-years, 3-years, or 5-years and beyond. The pooled HR for 2-year estimation was 0.44 [0.13, 0.76], *p* = 0.006, which supports better survival achieved with cisplatin-based therapy, while the 3-year or 5-year and beyond time assessments showed no significant difference between the two groups, with pooled HRs of 0.21 [-0.14, 0.55], *p* = 0.241and 0.95 [0.51, 1.74], *p* = 0.86, respectively (Table 2; Fig. 2).

**Fig. 3:**
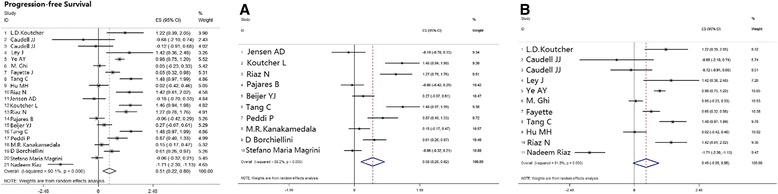
Meta-analysis compared cisplatin-based chemoradiotherapy versus cetuximab-based bioradiotherapy in estimating patients in HPV+ subgroup regarding to OS, PFS, and LRC. OS, overall survival; PFS, progression-free survival; LRC, locoregional control

Human papillomavirus (HPV) infection state might contribute to pathogenesis of HNSCC, and it has previously been demonstrated that HPV positive (HPV+) cases showed better prognosis and prolonged survival in the cetuximab single agent group. The pooled HR is 1.12 [0.46, 2.17], *p* = 0.015 (Table 2; Fig. 3).

**Fig. 4:**
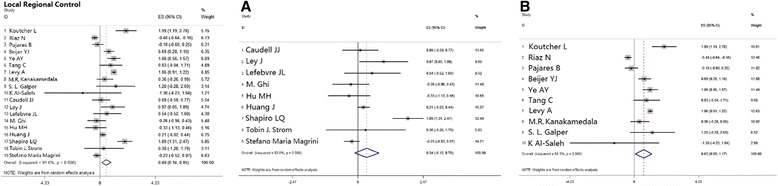
Meta-analysis compared cisplatin-based chemoradiotherapy versus cetuximab-based bioradiotherapy in estimating patients with oropharyngeal primary tumor regarding to OS, PFS and LRC. OS, overall survival; PFS, progression-free survival; LRC, locoregional control

The oropharynx was shown to be distinct in prognosis and therapy response compared with HNSCC in other locations. On this account, we analyzed this group separately, and the results showed that patients with primary tumors in the oropharynx exhibited similar values of OS with a pooled HR of 0.13 [-0.03, 0.89], *p* = 0.743 (Table 2; Fig. 4).

### Comparison between cisplatin-based and cetuximab therapies regarding progression-free survival

**Fig. 5:**
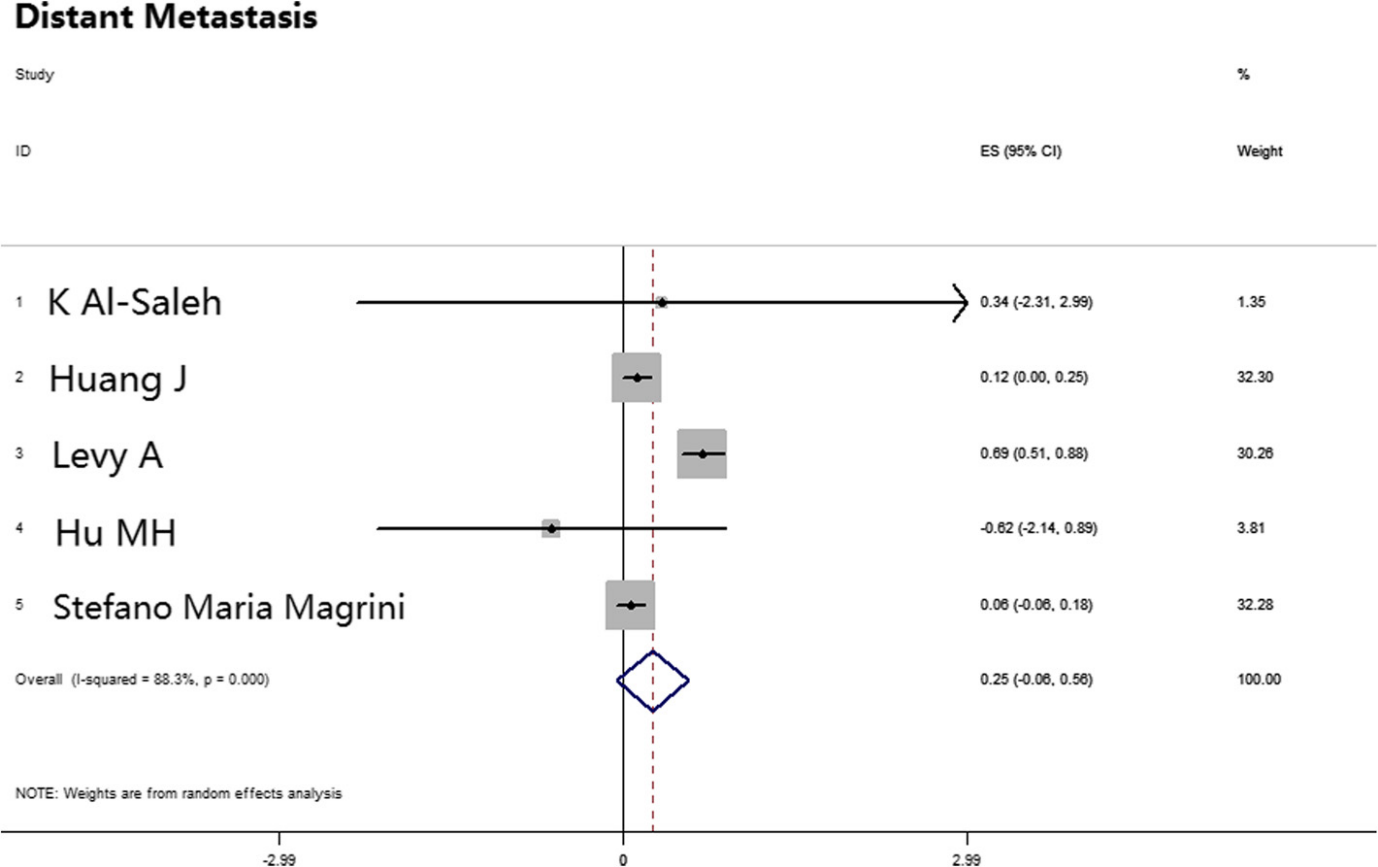
Meta-analysis estimated PFS comparing cisplatin-based chemoradiotherapy versus cetuximab-based bioradiotherapy. (**a**) subgroup of estimation of 2-yr PFS; (**b**) subgroup of estimation of 3-yr PFS. PFS, progression-free survival

Twenty-one studies published data including progression free survival (PFS). The PFS results displayed a similar tendency as the OS and the mathematic value for the pooled HR was 0.51 [0.22, 0.80], *p* = 0.001 (Table 2; Fig. 5).

#### Subgroup analysis

As assessed in the OS data, we categorized PFS outcomes by time intervals of estimation: 2-years, or 3-years and beyond. The pooled HRs were 0.56 [0.20, 0.92], *p* = 0.002, and 0.45 [-0.05, 0.95], *p* = 0.076 for the 2-year and 3-years and beyond time assessments, respectively, which indicate that better survival was achieved with cisplatin-based therapy (Table 2; Fig. 5).

For the HPV+ group, cetuximab-based therapy again showed outcomes superior to those of cisplatin-based therapy, and the pooled HR was 0.80 [0.38, 1.67], *p* = 0.55 (Table 2; Fig. 3).

We also analyzed PFS separately in patients with oropharynx tumors, and those patients who received cetuximab-based regimens showed prolonged PFS compared with administration of cisplatin-based therapy; the pooled HR was 1.56 [1.14, 2.13], *p* = 0.006 (Table 2; Fig. 4).

### Comparison between cisplatin-based and cetuximab therapies regarding loco-regional containment

**Fig. 6:**
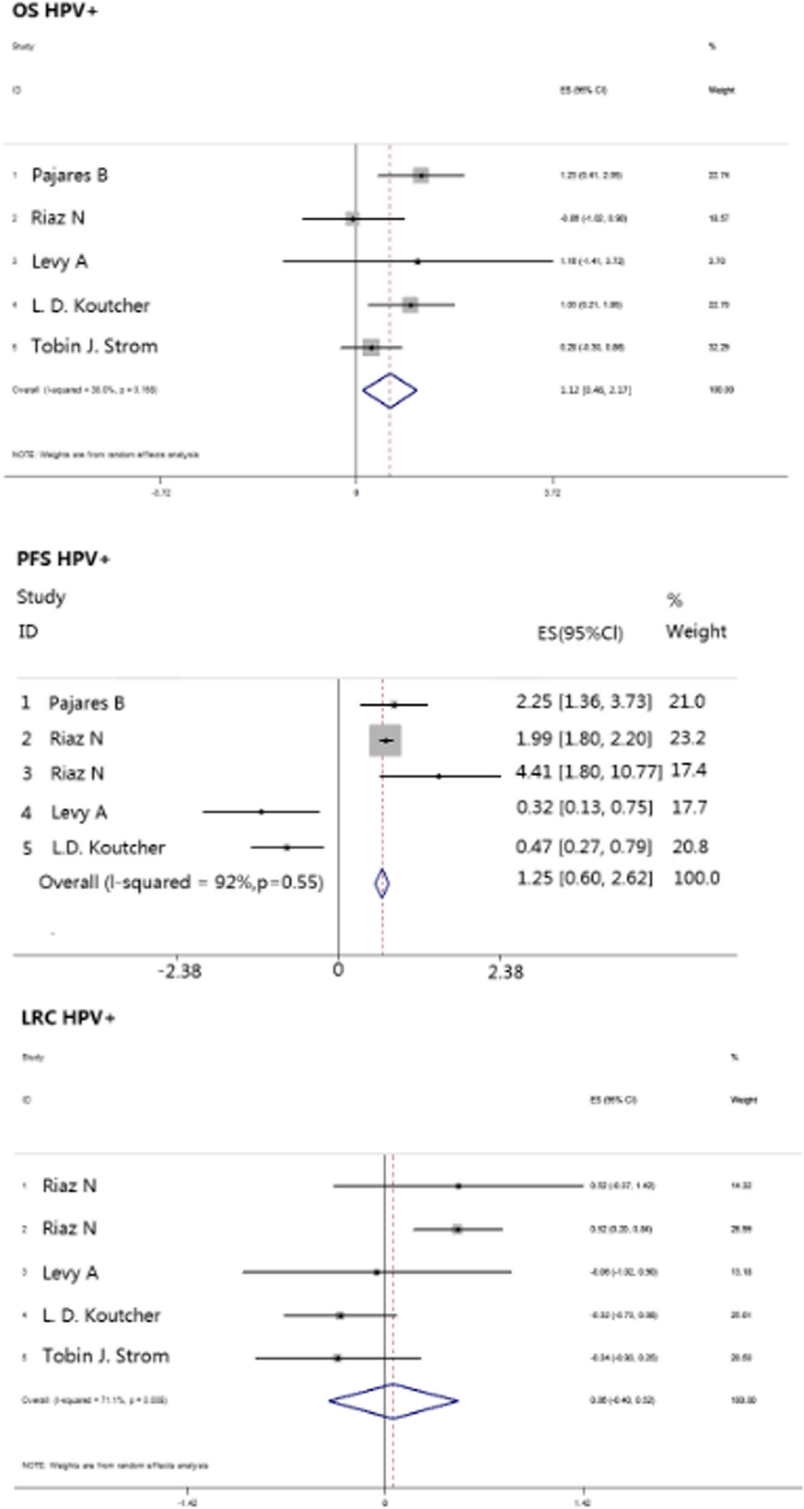
Meta-analysis estimated LRC comparing cisplatin-based chemoradiotherapy versus cetuximab-based bioradiotherapy. (**a**) subgroup of estimation of 2-yr LRC; (**b**) subgroup of estimation of 3-yr LRC. LRC, locoregional control

Nineteen studies reported loco-regional control or loco-regional failure in patients with HNSCC. The pooled HR to compare OS between the two groups showed better outcomes with cisplatin-based therapy, and the mathematic value was 0.49 [0.14, 0.85], *p* = 0.007 (Table 2; Fig. 6).

#### Subgroup analysis

Loco-regional control, like other recurrence rates and survival outcomes, directly correlated to the estimated time interval, and thus, we categorized the loco-regional control rates by the year of estimation: 2-years, 3-years, or 5-years and beyond. The pooled HR for the 2-year estimation was 0.63 [0.09, 1.17], *p* = 0.023, which supports better survival achieved with cisplatin-based therapy, while the 3-years or 5-years and beyond time assessments showed no significant difference between the two groups, and the pooled HRs were 0.34 [-0.12, 0.79], *p* = 0.15 and 2.67 [0.47, 8.73], *p* = 0.27, respectively (Table 2; Fig. 6).

Patients with HPV+ infection states showed a non-significantly better prognosis and prolonged survival in the cetuximab single agent group, and the pooled HR was 0.06 [-0.40, 0.52], *p* = 0.808 (Table 2; Fig. 3).

Analysis of patients with primary tumors in the oropharynx showed no significant difference between the cisplatin and cetuximab groups with a pooled HR of -0.05 [-1.34, 0.35], *p* = 0.248 (Table 2; Fig. 4).

### Comparison between cisplatin-based and cetuximab therapies regarding distant metastasis

**Fig. 7:**
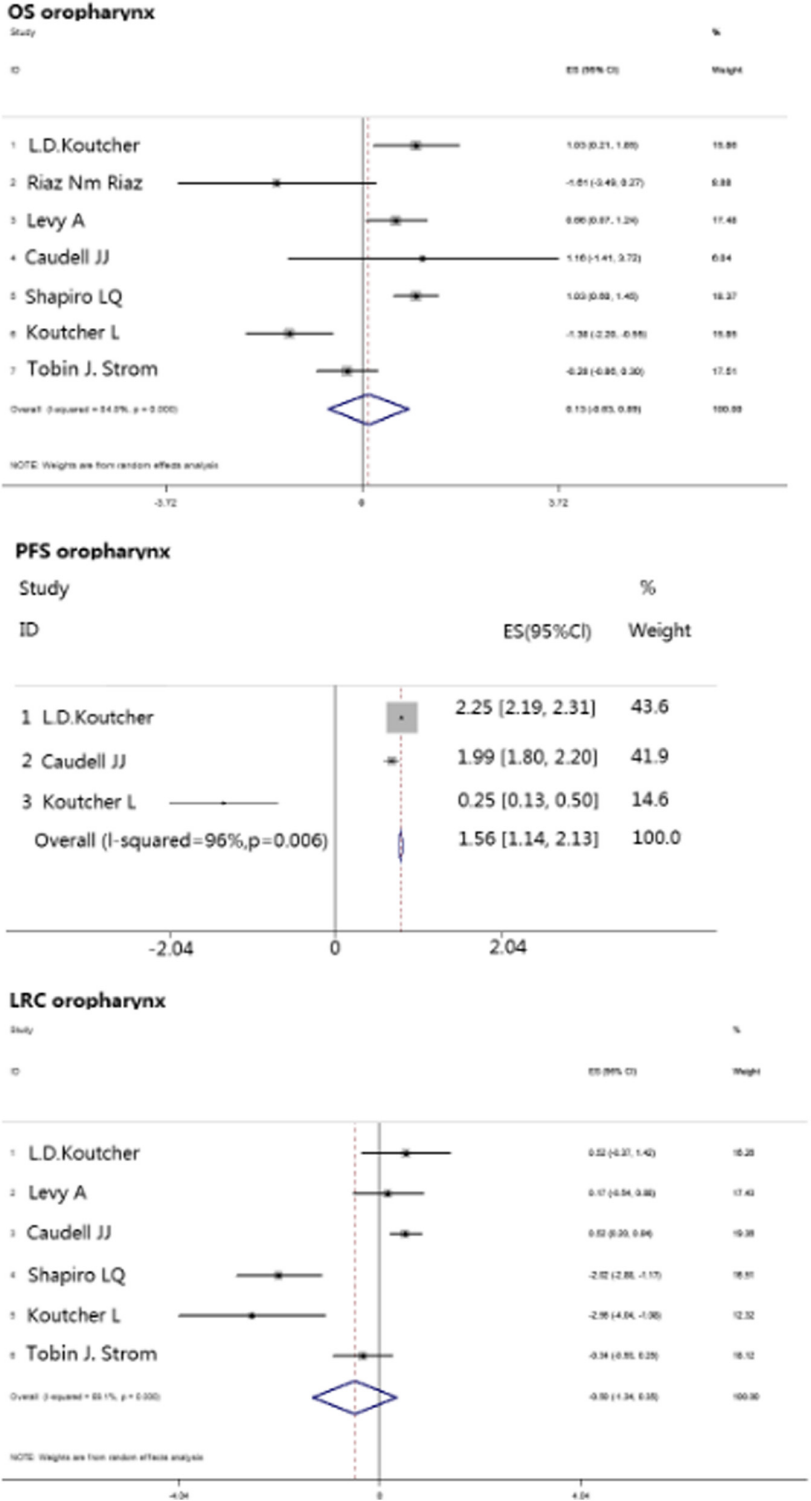
Meta-analysis estimated DM comparing cisplatin-based chemoradiotherapy versus cetuximab-based bioradiotherapy. DM, distant metastasis

Five studies reported incidences of distant metastases. The pooled HR was 0.25 [0-0.06, 0.56], *p* = 0.118, indicating no significant difference between cisplatin and cetuximab administration (Table 2; Fig. 7).

### Assessment of adverse events

**Fig. 8:**
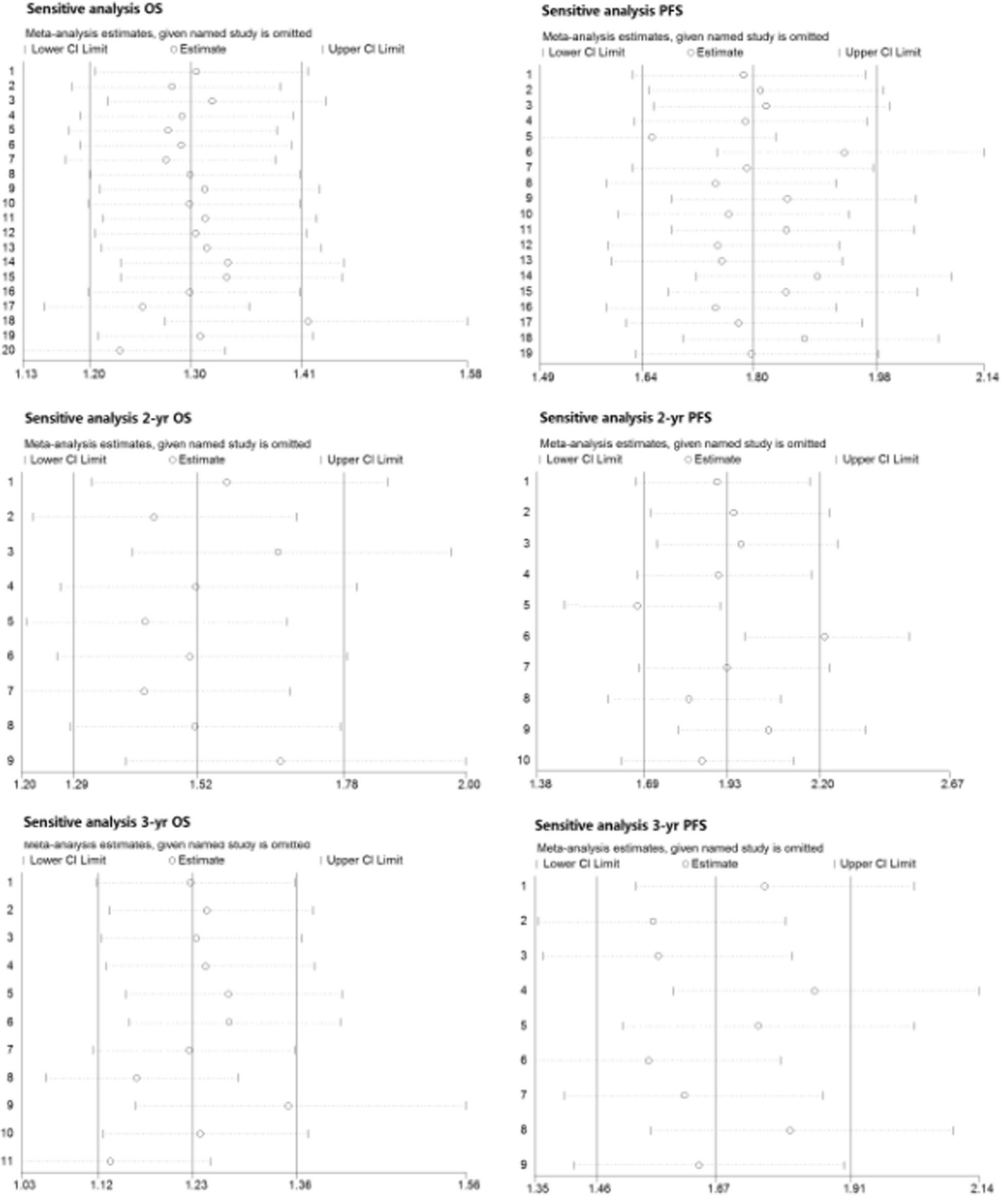
Meta-analysis compared cisplatin-based chemoradiotherapy versus cetuximab-based bioradiotherapy in estimating toxicities including acute and late toxicities

Twenty-one types of acute toxicity or late toxicity in patients treated with cisplatin plus radiotherapy or cetuximab plus radiotherapy were assessed. The pooled HRs of all toxicities, including acute and late toxicities, showed no difference for patients who received cisplatin-based or cetuximab-based therapy, and the mathematic value was -0.34 [-0.72, 0.04], *p* = 0.079 (Fig. 8).

#### Subgroup analysis

**Fig. 9:**
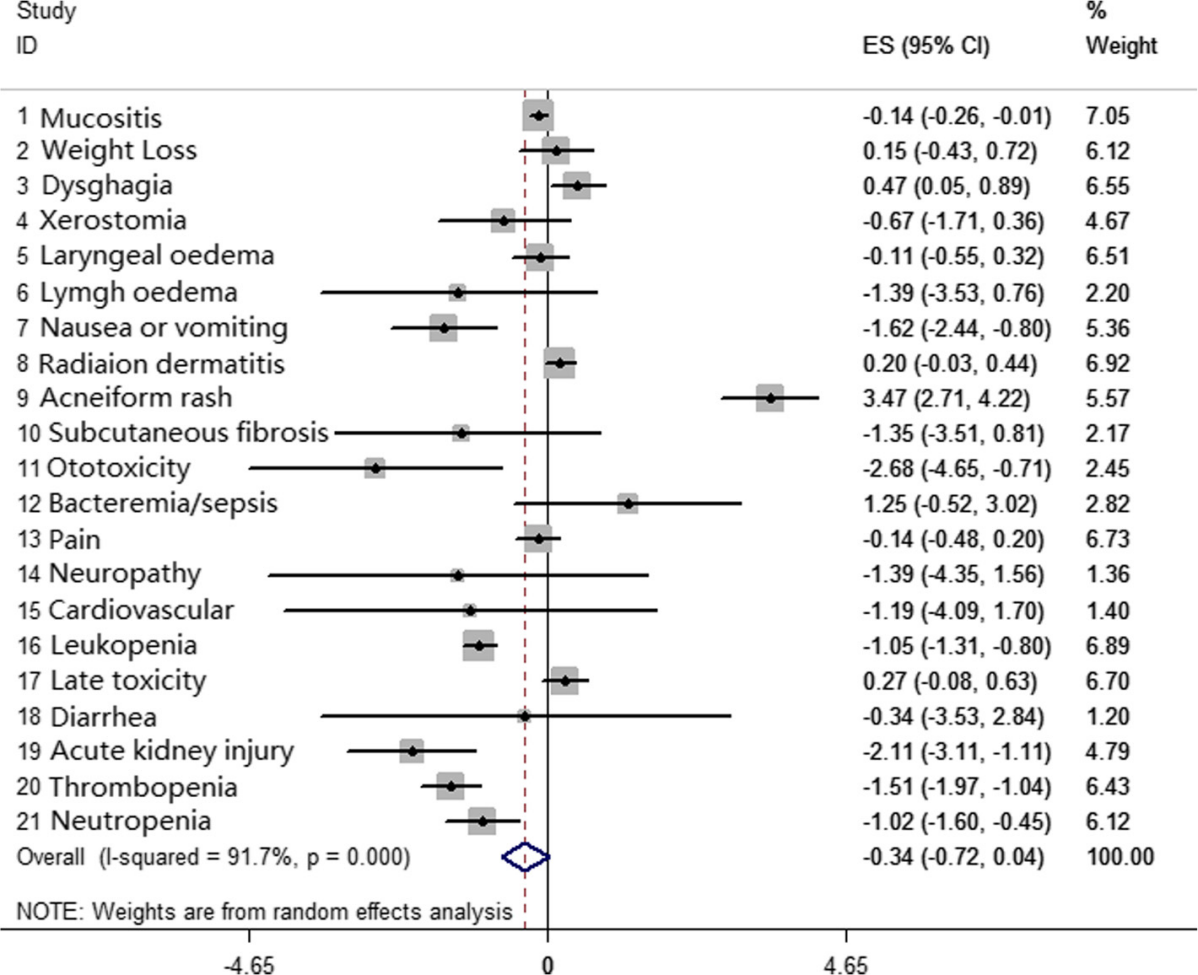
Meta-analysis compared cisplatin-based chemoradiotherapy versus cetuximab-based bioradiotherapy in estimating patients with oropharyngeal primary tumor and with single toxicity separately

We estimated individual toxicities separately, which is shown in Fig. 9. We found that incidence of toxicities, such as leukopenia (*p* = 0.00), acute kidney injury (*p* = 0.002), and neutropenia (*p* = 0.002), were significantly higher in the cisplatin plus radiotherapy regimen, while some dermatitis-related toxicities, such as acneiform rash (*p* = 0.002), displayed a higher incidence in the cetuximab plus radiotherapy regimen. Other toxicities showed no statistical significance between the two groups (Table 3; Fig. 9).

**Table 3:** Pooled HRs 95 % Cl for toxicity between CRT & BRT

Comparison	Adverse event/ Toxicity	Study N.	Model	HR 95 % [Cl]	*P* value	Heterogeneity (p ,I^2^)	Conclusion
CRT vs. BRT	Mucositis	7	Fixed	0.05 [-0.09, 0.19]	*p*=0.493	*P* = 0.45; I² = 36.9 %	Negative
CRT vs. BRT	Dysphagia	5	Fixed	-0.07 [-0.35, 0.21],	*p*=0.63	*P* = 0.89; I² = 0 %	Negative
CRT vs. BRT	Xerostomia	2	Fixed	0.51 [0.09, 2.95],	*p*=0.46	*P* = 0.17; I² = 46 %	Negative
CRT vs. BRT	Laryngeal edema	2	Fixed	0.91 [0.71, 1.18]	*p*=0.49	*P* = 0.89; I² = 0 %	Negative
CRT vs. BRT	Acute kidney injury	5	Fixed	-1.30 [-2.11, -0.49]	*p*=0.002	*P* = 0.32; I² = 0 %	Positive
CRT vs. BRT	Nausea or vomiting	4	Random	-1.30 [-2.66, 0.06],	*p*=0.061	*P* = 0.03; I² = 57.2 %	Negative
CRT vs. BRT	Radiation dermatitis	4	Random	0.31 [-0.45, 1.08]	*p*=0.419	*P* = 0.001; I² = 87.6 %	Negative
CRT vs. BRT	Acneiform rash	5	Random	3.49 [1.23, 5.74]	*P*=0.002	*P* = 0.87; I² = 81 %	Positive
CRT vs. BRT	Neutropenia	3	Fixed	-0.88 [-1.42, -0.33]	*p*=0.002	*P* < 0.00001; I² = 0.0 %	Positive
CRT vs. BRT	Ototoxicity	3	Fixed	0.16 [0.04, 0.69]	*p*=0.10	*P* = 0.60; I² = 0 %	Negative
CRT vs. BRT	Infectious	2	Fixed	3.31 [0.55, 19.87]	*p*=0.19	*P* = 0.59; I² = 0 %	Negative
CRT vs. BRT	Neuropathy	2	Fixed	0.80 [0.46, 1.41]	*p*=0.44	*P* = 0.37; I² = 0 %	Negative
CRT vs. BRT	Pain	2	Fixed	0.92 [0.80, 1.06]	*p*=0.24	*P* = 0.74; I² = 0 %	Negative
CRT vs. BRT	Leukopenia	4	Fixed	-0.76 [-1.16, -0.36]	*P*=0.001	*P* = 0.19; I² = 44.2 %	Positive
CRT vs. BRT	Late toxicity	4	Fixed	1.11 [0.83, 1.47],	*p*=0.48	*P* = 0.53; I² = 0 %	Negative
CRT vs. BRT	Total toxicity	21	Random	-0.34 [-0.72, 0.04]	*P*=0.079	*P* < 0.00001; I² = 91.7 %	Negative

*CRT* cisplatin-based chemoradiotherapy, *BRT* cetuximab-based bioradiotherapy, *N* number, *CI* confidence interval, *HR* hazard ratio, *CRT* chemoradiothrapy, *BRT* bioradiothrapy

### Results from sensitive tests

**Fig. 10:**
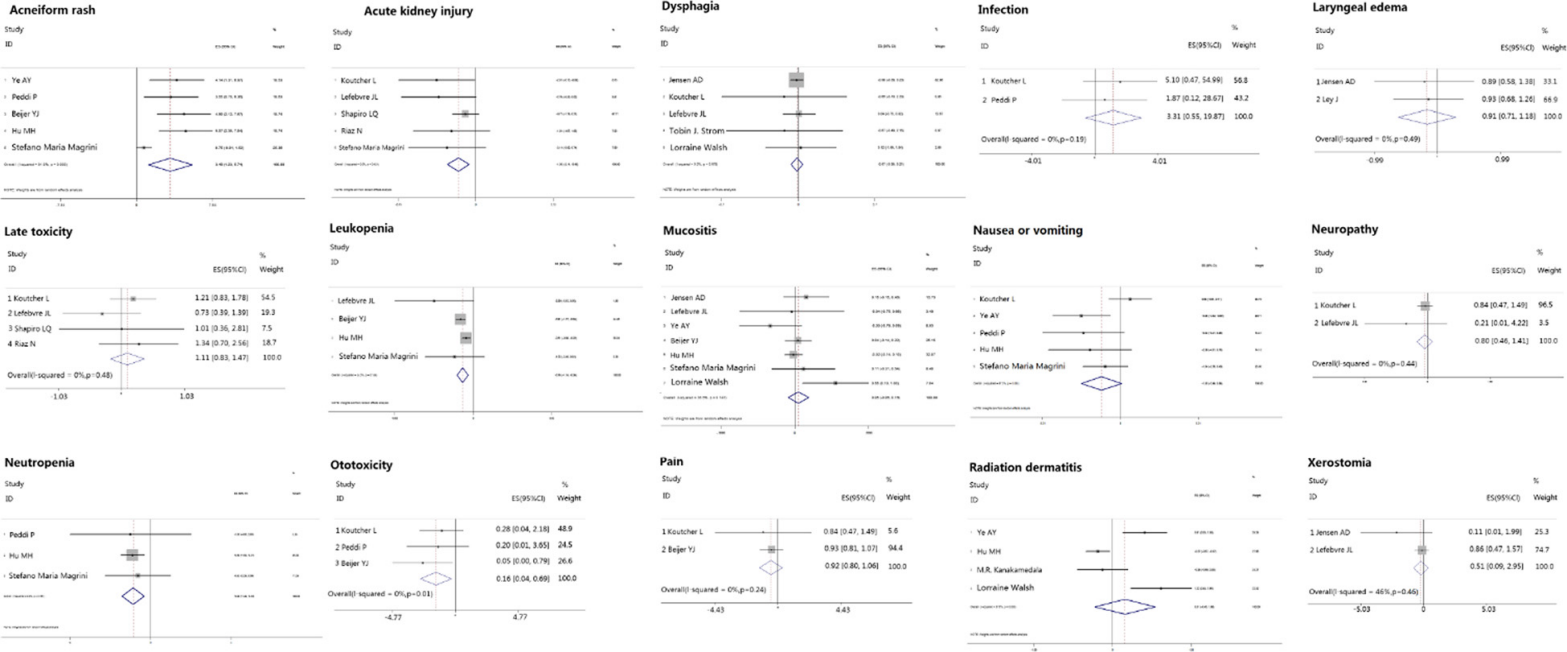
Sensitive analysis evaluated heterogeneity of OS cohort, PFS cohort and related subgroups. (**a**) Evaluation in OS group: total, 2-yr and 3-yr. (**b**) Evaluation in PFS group: total, 2-yr, and 3-yr. OS, overall survival; PFS, progression-free survival

As shown in Fig. 10, all of the scattered points were restricted within the interval of the lower CI and upper CI limitations, which indicated that the heterogeneity was acceptable and constrained (Fig. 10).

### Assessment of publication bias

**Fig. 11:**
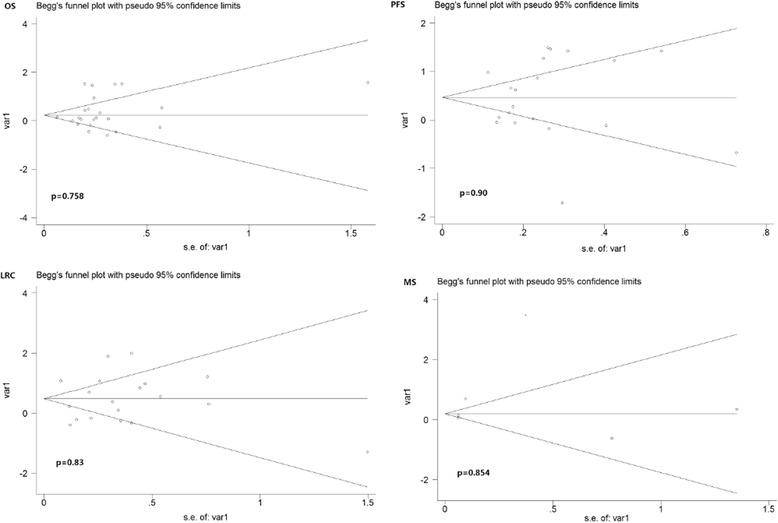
Estimated Begg’s funnel plots of publication bias regarding OS, PFS, LRC, and DM cohort respectively. OS, overall survival; PFS, progression-free survival; LRC, locoregional control; DM, distant metastasis

On the basis of Begg’s funnel plot, the *p* value was greater than 0.10, which indicates that the publication bias was acceptable in the analysis. According to Begg’s funnel plot analysis, the publication bias arising in the OS cohort (*p* = 0.758), the PFS cohort (*p* = 0.90), the loco-regional control cohort (*p* = 0.83) or the distant metastasis cohort (*p* = 0.854) was acceptable (Fig. 11).

## Discussion

In this systemic review, we conducted a meta-analysis to compare the effect of cisplatin-based chemotherapy plus radiotherapy versus cetuximab plus radiotherapy in controlling the overall survival, progression-free survival, loco-regional recurrence and distant metastasis of locally advanced HNSCC. Meanwhile, different time periods of estimation, primary tumor sites in the oropharynx and HPV infection status were also taken into consideration. Our study demonstrated that in all settings of the estimated OS time duration, the outcomes were found to be better with cisplatin treatment; however, specifically observing the longer follow-up time intervals, patients between the two groups shared similar overall survival rates, as there was no statistical significance between the two groups with a follow-up time duration equal to or longer than 3 years. Progression-free survival and loco-regional control rates displayed similar tendencies as the OS rates. In subgroup analysis, tumors with a primary site in the oropharynx and tumors with HPV+ infection status showed non significantly better PFS and OS, respectively, with cetuximab single agent treatment plus radiotherapy, while no remarkable difference was observed between the remaining survival outcomes and loco-regional control in the two subgroups, indicating that equivalent effects of the two treatment regimens were achieved in these categories.

Concurrent cisplatin-based therapy has been regarded as the standard treatment regimen for patients with HNSCC [51]; however, cisplatin has been reported to cause immediate treatment-related adverse events and delayed toxicity. Cetuximab, an emerging monoclonal antibody therapeutic, targeting epidermal growth factor receptor (EGFR), seemed promising to provide patients with an effective alternative treatment [52]. Whether cetuximab could replace cisplatin in definitive chemoradiotherapy for HNSCC remains controversial because cetuximab has a reasonably good toxicity profile [53] but the tumor control effect and survival benefit present inconsistent results.

Therefore, to achieve better quality of life and avoid these aggressive treatment regimens, concurrent cetuximab plus radiation versus cisplatin plus radiation therapies have been compared. A recent meta-analysis including 15 studies comparing CCRT and concurrent cetuximab with radiotherapy, with various estimation time intervals, suggested that cisplatin usage improved OS and PFS [54], consistent with our results. Nevertheless, it seems that these drug responses and effects will benefit patients in certain circumstances. In our analysis, we showed that patients from selected subgroups of HNSCC might benefit from concurrent cetuximab plus radiotherapy.

In our analysis, we found that both the oropharyngeal primary tumor and HPV+ subgroups showed differences regarding survival outcomes, possibly supporting the utility of cetuximab to a large extent.

We focused on HPV+ patients, as the biological behavior of these tumors showed particularity. HPV is now considered to be an independent and important risk factor in HNSCC [55, 56]. Recently, *Dayyani* et al. published a meta-analysis, which showed that HPV infection has a critical impact on survival and response to therapy, and they also demonstrated that HPV+ status was not rare (HPV+ 22 %, with 86.7 % exhibiting HPV16+ genotype) [57]. However, some negative outcomes also exist, and no significant difference was shown between cisplatin and cetuximab with radiation in LAHNC [58]. One major obstacle in this work was the lack of information regarding the HPV/p16 status; thus, we suggest that patients should undergo HPV testing for this unique and separate biologic entity. In our analysis, patients in the HPV+ group achieved better OS due to the highly selective and biologic characteristics, which made the HPV+ group more suitable for the concurrent BRT treatment regimen than the whole HNSCC group.

We also observed unique responses in patients who had primary lesions in the oropharynx. One comprehensive study estimated chemotherapy effects via tumor sites, and the results showed increased benefits only for oropharyngeal and laryngeal tumors [59, 60]. There are well-established patient risk factors associated with HPV infection in oropharyngeal cancer, and a higher incidence of HPV infection was found in cancers of the oropharynx [61, 62]. In our analysis, better PFS was observed in the oropharyngeal group rather than all cases of HNSCC, which could support the administration of cetuximab as a single agent plus radiation in this specific subgroup.

Adverse effects are important additional parameters to be taken into consideration when comparing treatment regimens. In our analysis, we found that there were no significant differences between the two groups for all toxicity data. The cisplatin regimen resulted in adverse events, including high-grade neutropenia, leukopenia and acute kidney injury, while adverse events due to BRT included grade 3-4 acne-like rash and oral mucositis. We found that the incidence of adverse events was elevated in advanced cases. One recent study published by *Lawrence D. Koutcher et al*, showed serious grade radiation dermatitis with spontaneous bleeding in patients undergoing the BRT regimen [63], which could further decrease quality of life [64] and have a negative impact on cosmetic outcomes [65]. As the total incidence of adverse events did not show significance between cisplatin and cetuximab and the two regimens cause different adverse events in different aspects, doctors need to take toxicity into consideration and choose regimens according to each patient’s condition.

To further confirm the quantity of evidence of the analyzed the data, heterogeneity and sensitive analysis were examined, and no obvious heterogeneity was detected; as shown in every group estimation, I^2^ was <50 %, with no exception. In addition, the further sensitive analysis assessed heterogeneity in detail and revealed only limited heterogeneity. In addition, as this is a meta-analysis, some limitations still exist. Primarily, only published data from prospective or retrospective studies were included in our meta-analysis, without individual data. Therefore, we could only use these integrated data, which may lead to patient selection bias as patient selection and reporting processes could not be controlled by us. In addition, we combined both retrospective and randomized trials in our meta-analysis, which could also contribute to the bias of this meta-analysis as the inclusion criteria of these two types of studies may not be the same and result in mixed data bias. Additionally, in pooled-data calculation processes, we chose multivariate data, if they were available. Otherwise, our calculated data consisted of univariate data without adjusting for some other influencing factors, such as age, sex, and histologic grade. This would represent a source of bias because multivariate studies examine the prognostic value independently, while univariate studies consider single factor.

## Conclusion

In spite of all of the limitations and biases of our meta-analysis, we conclude that long-term use of cetuximab plus radiation showed no significant difference compared with cisplatin plus radiation for all of the survival and toxicity data examined. In subgroup analysis, cetuximab plus radiation may show superior responses regarding OS and PFS in patients who have HPV+ or primary oropharyngeal HNSCC, respectively, but physicians should administer them with caution.

This analysis is a combination of current data. Previously, it was thought that cetuximab could cause fewer side effects and may be preferable to cisplatin, as they showed similar survival outcomes. However, we showed that the two regimens caused toxicity without significant differences, while cisplatin treatments exhibited better survival outcomes. Thus, with all of the limitations, we recommend further RCTs to determine the utility of cetuximab in HNSCC, especially in the oropharyngeal and/or HPV+ specific subgroups.
